# An essential role for the RNA helicase DDX6 in NMDA receptor-dependent gene silencing and dendritic spine shrinkage

**DOI:** 10.1038/s41598-024-53484-4

**Published:** 2024-02-06

**Authors:** Fathima M. Perooli, Kevin A. Wilkinson, Kate Pring, Jonathan G. Hanley

**Affiliations:** 1 School of Biochemistry, University of Bristol, University Walk, Bristol, BS8 1TD UK; 2 School of Physiology, Pharmacology and Neuroscience, University of Bristol, University Walk, Bristol, BS8 1TD UK

**Keywords:** Molecular neuroscience, Synaptic plasticity, Molecular biology, miRNAs, Neuroscience, Spine plasticity

## Abstract

MicroRNAs (miRNAs) repress translation of target mRNAs by associating with Argonaute (Ago) proteins in the RNA-induced silencing complex (RISC) to modulate protein expression. Specific miRNAs are required for NMDA receptor (NMDAR)-dependent synaptic plasticity by repressing the translation of proteins involved in dendritic spine morphogenesis. Rapid NMDAR-dependent silencing of *Limk1* is essential for spine shrinkage and requires Ago2 phosphorylation at S387. Not all gene silencing events are modulated by S387 phosphorylation, and the mechanisms that govern the selection of specific mRNAs for silencing downstream of S387 phosphorylation are unknown. Here, we show that NMDAR-dependent S387 phosphorylation causes a rapid and transient increase in the association of Ago2 with *Limk1*, but not *Apt1* mRNA. The specific increase in *Limk1* mRNA binding to Ago2 requires recruitment of the helicase DDX6 to RISC. Furthermore, we show that DDX6 is required for NMDAR-dependent silencing of *Limk1* via miR-134, but not *Apt1* via miR-138, and is essential for NMDAR-dependent spine shrinkage. This work defines a novel mechanism for the rapid transduction of NMDAR stimulation into miRNA-mediated translational repression of specific genes to control dendritic spine morphology.

## Introduction

To fulfil the brain’s function of information processing and storage, neurons undergo extensive long-lasting plasticity. Tightly regulated changes in synaptic transmission and the morphology of dendritic spines, which house synapses, underlie the formation and modification of neural circuits during brain development and memory processes^1,2^. Long-Term Depression (LTD) is defined as a decrease in synaptic strength with a corresponding decrease in dendritic spine size, whereas Long-Term Potentiation (LTP) is an increase in synaptic strength with a corresponding increase in spine size^3,4^. LTD and LTP are triggered primarily by stimulation of NMDA receptors (NMDARs) and underpin specific types of learning and memory processes in corresponding brain regions^5,6^. Long-term plasticity involves complex cellular responses including changes in the local proteome close to synapses by regulating mRNA translation^7,8^.

MicroRNAs (miRNAs) repress gene expression by recruiting Argonaute (Ago) proteins and the RNA-induced silencing complex (RISC) to 3’UTRs of target mRNAs by complementary base pairing. They are fundamentally important in a wide range of cellular processes for fine-tuning localised protein synthesis^9,10^. Numerous specific miRNAs are essential for long-term neuronal plasticity and consequent memory formation by regulating the translation of key synaptic proteins^8,11,12^. We and others have shown previously that the NMDAR-dependent activation of miRNA-dependent gene silencing is required to modulate the expression of proteins that regulate the actin cytoskeleton or membrane trafficking processes to cause changes in spine morphology and induce LTD^13–16^.

Depletion of specific miRNAs blocks plasticity expressed just a few minutes after stimulation and blocks the formation of memories that take a similarly short period of training, indicating that miRNA systems are critical for such rapid plasticity^14,17–19^. While NMDAR stimulation regulates the transcription of miRNA genes, it takes 1 h following stimulus to reach a significant increase in mature miRNA^14,15^. Furthermore, individual miRNAs typically target several different mRNAs^10,20^; therefore, increasing transcription of an individual miRNA does not provide the necessary silencing specificity on its own. Hence, a major gap in our understanding is how specific subpopulations of mRNAs are rapidly selected for miRNA-dependent silencing to cause localised changes to the proteome for plasticity. We propose that alternative NMDAR-dependent mechanisms, which rely on existing pools of available miRNAs and are not constrained by the time taken to transcribe miRNAs and transport them along dendrites, must be employed to cause rapid silencing.

We recently demonstrated that NMDAR-dependent Ago2 phosphorylation at S387 is required for NMDAR-dependent shrinkage of dendritic spines by rapidly (< 10 min after stimulation) increasing miR-134-dependent silencing of *Limk1*, which encodes the actin-regulatory protein Lim kinase 1^16^. S387 phosphorylation did not affect silencing of other miR-134 targets, indicating that the specificity for this mechanism lies in the 3’UTR of the target mRNA. Furthermore, NMDAR stimulation also enhanced miR-138 dependent silencing of the Acyl-protein thioesterase *Apt1/Lypla1*^16,21^, which regulates palmitoylation of synaptic proteins and is involved in synaptic plasticity^22,23^. Both miR-134 and miR-138 have been shown previously to regulate dendritic spine morphology^24^. While silencing of *Apt1* was enhanced by NMDAR stimulation, this was not mediated by Ago2 phosphorylation at S387, suggesting the existence of distinct populations of genes silenced by diverging mechanisms in response to neuronal signalling.

RISC comprises several core proteins and interacts with accessory proteins with roles in regulating RNA processing, stability or translation, providing strong potential for functional regulation in response to cell signalling pathways^25,26^. The RNA helicase DDX6 is emerging as an important accessory protein for RISC function^27,28^, and associates with RISC via direct binding to CNOT1 of the CCR4-NOT complex, which in turn binds Ago2 via the scaffolding protein GW182^29–31^. NMDAR-dependent S387 phosphorylation of Ago2 increases its association with DDX6^16,32^, leading to the hypothesis that DDX6 is involved in the S387 phosphorylation-dependent increase in silencing of specific genes. While the regulation of miRNA activity by DDX6 has been studied in the context of neuronal differentiation^33^, a role for DDX6 in neuronal plasticity has not been reported previously.

Here, we identify a mechanism for the recruitment of specific mRNAs to RISC within a few minutes after NMDAR stimulation that is mediated by RISC-associated DDX6. Furthermore, we demonstrate that enzymatically active DDX6 is required for NMDAR-dependent dendritic spine shrinkage.

## Results

### NMDAR-dependent S387 phosphorylation increases Ago2 interaction with DDX6 protein and *Limk1* mRNA.

Our previous work demonstrated that silencing of *Limk1* by miR-134 is modulated by Ago2 phosphorylation at S387, whereas *Apt1* silencing by miR-138 is unaffected by S387 phosphorylation^16^. Hence, in the current study, we investigate *Limk1* and *Apt1* as representatives of populations of genes regulated by, or unaffected by S387 phosphorylation of Ago2, respectively.

**Figure 1:**
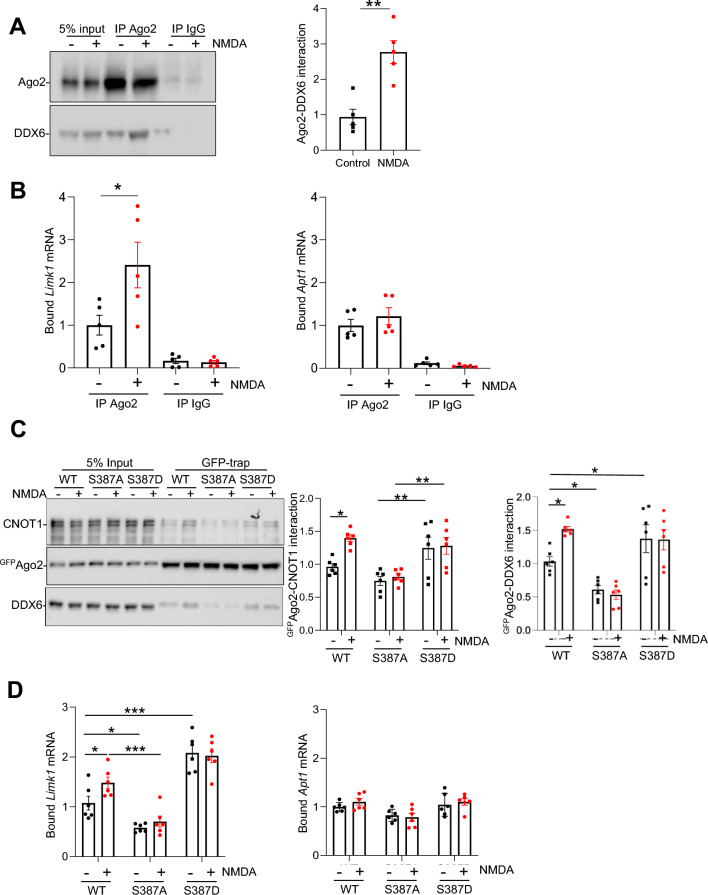
NMDAR stimulation increases the association of *Limk1*, but not *Apt1* mRNA with Ago2 via S387 phosphorylation. (**A** and **B**) Cultured neurons were stimulated with NMDA or vehicle for 3 min, followed by 10 min incubation after NMDA washout. Cells were lysed and subjected to immunoprecipitation with anti-Ago2 or control IgG. RNA and proteins were isolated by trizol extraction, bound mRNAs were quantified by qPCR and proteins were detected by Western blotting. (**A**) NMDAR stimulation increases Ago2-DDX6 interaction. Left panels show representative Western blots, graph shows quantification of DDX6-Ago2 interaction in response to NMDAR stimulation. n = 5 independent experiments, **p < 0.01, t-test. Data are mean ± S.E.M. (**B**) NMDAR stimulation increases binding of *Limk1*, but not *Apt1* mRNA to Ago2. Graphs show quantification of *Limk1* and *Apt1* mRNA bound to Ago2 or to IgG control. Values are IP/input ratios, normalised to vehicle control. n = 5 independent experiments, *p < 0.05, two-way ANOVA followed by Tukey’s multiple comparison test. Data are mean ± S.E.M. (**C** and **D**) Neurons transduced with lentiviral vectors expressing ^GFP^Ago2(WT, S387A, or S387D) were stimulated with NMDA or vehicle for 3 min, followed by 10 min incubation after NMDA washout, lysed and incubated with GFP-trap beads. RNA and proteins were isolated by trizol extraction, bound mRNAs were quantified by qPCR and proteins were detected by Western blotting using anti-GFP, anti-CNOT1 and anti-DDX6. (**C**) NMDAR-dependent increase in DDX6 and CNOT1 interactions with Ago2 are mediated by S387 phosphorylation. Left panel shows representative Western blots, right panels show quantification of ^GFP^Ago2-DDX6 and ^GFP^Ago2-CNOT1 interactions, normalised to WT vehicle condition. n = 4 independent experiments, **p < 0.01, ***p < 0.001, two-way ANOVA followed by Tukey’s multiple comparison test. Data are mean ± S.E.M. (**D**) NMDAR-dependent increase in *Limk1* mRNA binding to Ago2 is mediated by S387 phosphorylation. Graphs show quantification of *Limk1* and *Apt1* mRNA bound to ^GFP^Ago2. Values are IP/input ratios, normalised to WT vehicle control condition. n = 5 independent experiments, *p < 0.05, **p < 0.01. ***p < 0.001, two-way ANOVA followed by Tukey’s multiple comparison test. Data are mean ± S.E.M.

To investigate the mechanism that underlies the selective silencing of *Limk1*, but not *Apt1*, by NMDAR-dependent S387 phosphorylation of Ago2 in neurons, we hypothesised that NMDAR stimulation increases the association of Ago2 with *Limk1* mRNA. To test this, we stimulated neuronal cultures for 3 min with NMDA and carried out Ago2 RNA-immunoprecipitations (RIPs) 10 min later, using Ago2-specific antibodies to isolate endogenous Ago2-containing protein complexes and associated RNA from cell lysates. Figure 1A demonstrates efficient IP of Ago2, and also confirms the NMDAR-dependent increase in Ago2-DDX6 interaction that we reported previously^16^. RT-qPCR analysis demonstrated that NMDAR stimulation caused a significant increase in binding of *Limk1* mRNA with endogenous Ago2 (Fig. 1B). In contrast, binding of *Apt1* mRNA to Ago2 was unaffected by NMDAR stimulation (Fig. 1B). To test the hypothesis that the NMDAR-dependent increase in *Limk1* mRNA binding to Ago2 is caused by S387 phosphorylation, we expressed ^GFP^Ago2(WT), a phospho-null mutant ^GFP^Ago2(S387A) or a phospho-mimic ^GFP^Ago2(S387D) in cultured neurons and stimulated with NMDA. These constructs were described and characterised previously^16^. We used GFP-trap beads to isolate the recombinant proteins, and analysed protein complexes by Western blotting and bound mRNAs by qPCR. Figure 1C demonstrates efficient isolation of recombinant ^GFP^Ago2, confirms the S387 phosphorylation-dependent increase in Ago2-DDX6 interaction^16^, and further demonstrates that CNOT1, which binds DDX6 directly^29–31^, associates with Ago2 in an NMDAR and pS387-dependent manner. In agreement with our hypothesis, the phospho-null mutation (S387A) caused a significant decrease in *Limk1* mRNA binding compared to Ago2(WT) under basal conditions, whereas the phospho-mimic (S387D) caused a significant increase (Fig. 1D). In response to NMDAR stimulation, ^GFP^Ago2(WT) showed an NMDAR-dependent increase in binding to *Limk1* mRNA, which was completely blocked by S387A and occluded by S387D (Fig. 1D). In contrast, neither NMDAR stimulation nor S387 mutation significantly affected *Apt1* mRNA binding to Ago2 (Fig. 1D). Taken together, these results demonstrate that NMDAR stimulation causes an increase in binding of *Limk1*, but not *Apt1,* mRNA with Ago2, and that phosphorylation of Ago2 at S387 is a necessary component of this mechanism.

**Figure 2:**
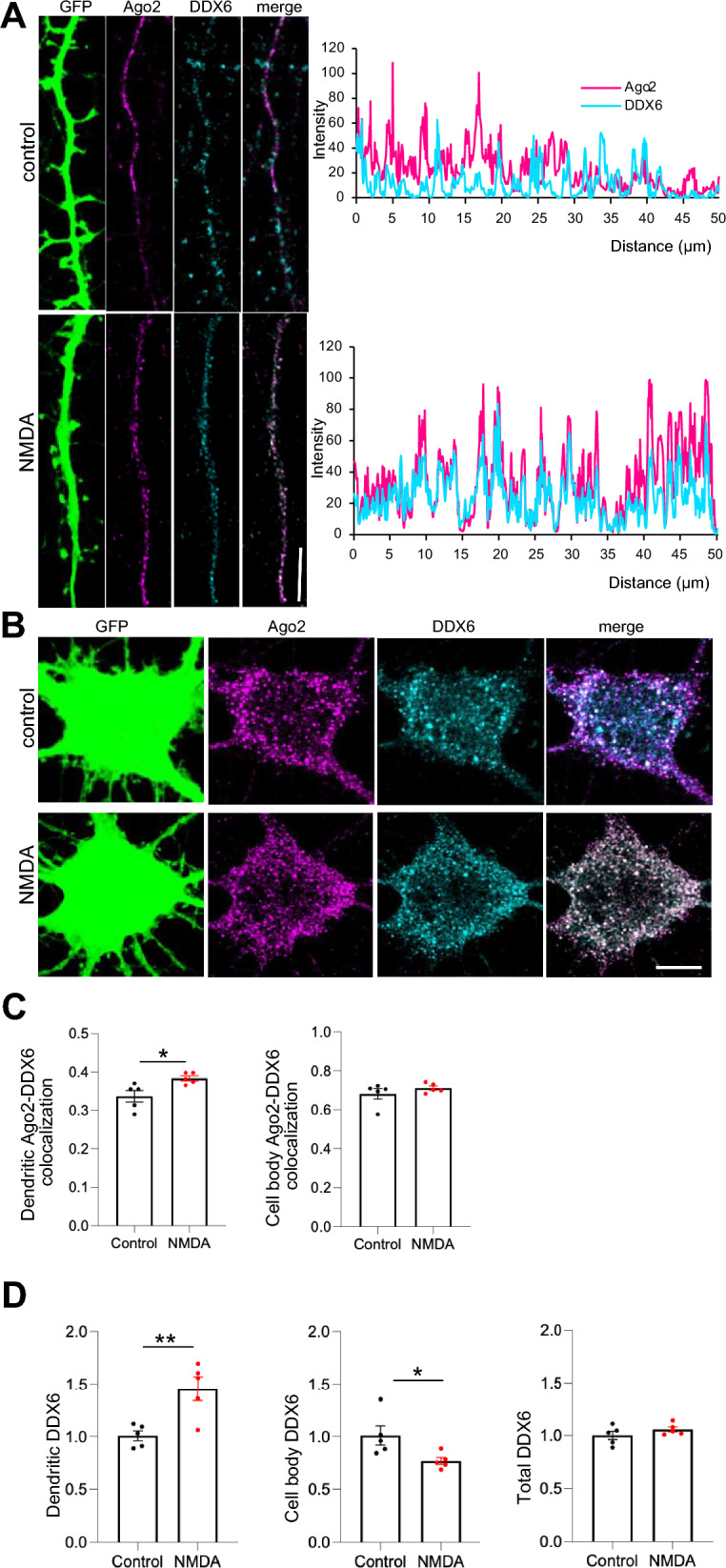
NMDAR-dependent increase in dendritic DDX6 and colocalization with Ago2. Cultured neurons were treated with NMDA or vehicle for 3 min. 10 min after NMDA washout, cells were fixed, permeabilised and stained with anti-Ago2 (magenta) and anti-DDX6 (cyan). (**A**) Ago2-DDX6 colocalisation in dendrites increases after NMDAR stimulation. Representative images of dendrites are shown; scale bar = 10 μm. Graphs show line-scan analyses of Ago2 and DDX6 fluorescence intensities in control and NMDA-stimulated dendrites shown in the left panels. (**B**) Ago2-DDX6 colocalisation in neuronal cell bodies is unaffected by NMDAR stimulation. Representative images of cell bodies are shown; scale bar = 5 μm. (**C**) Graphs show Pearson’s colocalisation coefficients for dendritic (left) and cell body (right) Ago2-DDX6 colocalisation. n = 5 independent experiments (4–6 cells per experiment), *p < 0.05, t-test. (**D**) Graphs show quantification of DDX6 expression level in dendrites (left) cell bodies (centre) and whole cell (right). n = 4 independent experiments (4–6 cells per experiment), *p < 0.05, **p < 0.01, t-test.

To investigate the NMDAR-dependent association of DDX6 with RISC further, we analysed the subcellular colocalization of endogenous DDX6 and Ago2 in cultured neurons by immunocytochemistry. Consistent with an increased biochemical interaction, colocalization of Ago2 and DDX6 was significantly increased by NMDAR stimulation in neuronal dendrites (Fig. 2A,C), while no change was observed in cell bodies (Fig. 2B,C). We also observed a significant increase in the dendritic localization of DDX6, with a concomitant decrease in cell body localized DDX6. This appears to represent protein translocation from cell body to dendrites, since there was no change in total DDX6 after NMDAR stimulation (Fig. 2D).

### DDX6 mediates NMDAR/pS387-dependent increase in binding of *Limk1* mRNA to Ago2

**Figure 3:**
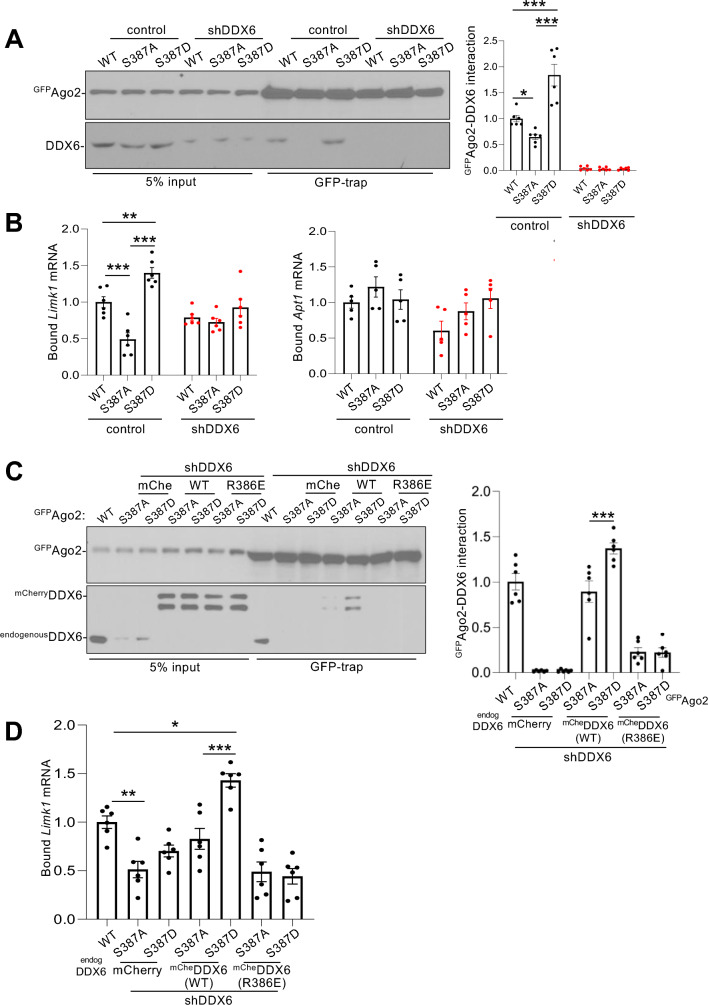
RISC-associated DDX6 is necessary for pS387-dependent increase in binding of *Limk1* mRNA to Ago2. (**A**) DDX6 knockdown abolishes S387 phosphorylation-dependent *Limk1* mRNA binding to Ago2. Neurons transduced with lentiviral vectors expressing ^GFP^Ago2(WT, S387A or S387D) and mCherry with or without DDX6 shRNA as shown were lysed and incubated with GFP-trap beads. RNA and proteins were isolated by trizol extraction, bound mRNAs were quantified by qPCR and proteins were detected by Western blotting using anti-GFP or anti-DDX6 antibodies. Representative Western blots are shown. Graph shows quantification of endogenous DDX6 bound to ^GFP^Ago2. n = 5 independent experiments, *p < 0.05, **p < 0.01, two-way ANOVA followed by Tukey’s multiple comparison test. Data are mean ± S.E.M. (**B**) Graphs show quantification of *Limk1* and *Apt1* mRNA bound to ^GFP^Ago2. Values are IP/input ratios, normalised to WT control condition. n = 5 independent experiments, *p < 0.05, **p < 0.01, ***p < 0.001, two-way ANOVA followed by Tukey’s multiple comparison test. Data are mean ± S.E.M. (**C**) Disrupting DDX6 association with RISC abolishes S387 phosphorylation-dependent DDX6 binding to Ago2 and *Limk1* mRNA binding to Ago2. Neurons transduced with lentiviral vectors expressing ^GFP^Ago2(WT, S387A or S387D) and mCherry, DDX6 shRNA or ^mCherry^DDX6(WT or R386E) as shown were treated as in (**A**). Left panel shows representative blots, graph shows quantification of endogenous DDX6 (lane 1) or ^mCherry^DDX6 binding to ^GFP^Ago2. n = 5 independent experiments, **p < 0.01, ***p < 0.001, one-way ANOVA followed by Tukey’s multiple comparison test. Data are mean ± S.E.M. (**D**) Graph shows quantification of *Limk1* mRNA bound to ^GFP^Ago2. Values are IP/input ratios, normalised to WT control condition. n = 6 independent experiments, *p < 0.05, **p < 0.01, one-way ANOVA followed by Tukey’s multiple comparison test. Data are mean ± S.E.M.

Since the association of DDX6 with Ago2 increases as a result of NMDAR-dependent S387 phosphorylation in neurons (Fig. 1;^16^), and S387 phosphorylation has a similar effect in non-neuronal cells^32^, we hypothesized that DDX6 plays a role in mediating the increase in *Limk1* mRNA binding to Ago2 caused by NMDAR-dependent S387 phosphorylation. To test this, we knocked down DDX6 expression by shRNA in neurons expressing ^GFP^Ago2(WT, S387A or S387D) and analysed bound mRNA by qPCR. The efficiency of DDX6 knockdown is shown in Fig. 3A and in Supplementary Fig. 1. Interestingly, DDX6 depletion completely blocked the phosphorylation-dependent binding of *Limk1* mRNA to ^GFP^Ago2 (Fig. 3B). In contrast, *Apt1* mRNA binding to Ago2 was not significantly affected by DDX6 knockdown (Fig. 3B).

DDX6 associates with RISC via a direct interaction with CNOT1 of the CCR4-NOT complex, which in turn binds GW182, the core scaffold protein that binds directly to Ago2^29–31^. We hypothesized that DDX6 must associate with RISC to mediate the S387 phosphorylation-dependent increase in *Limk1* mRNA binding to Ago2. It has been shown previously that a R386E mutation in DDX6 inhibits binding to CNOT1 and therefore to RISC^29^, so we generated lentiviral constructs to knock down endogenous DDX6 and simultaneously express shRNA-resistant ^mCherry^DDX6(R386E) or ^mCherry^DDX6(WT) (Fig. 3C and Supplementary Fig. 1). We co-expressed these constructs with ^GFP^Ago2(WT, S387A or S387D), and carried out Ago2 RIPs using GFP-trap beads. Western blot analysis confirmed the reduced association of Ago2 to DDX6(R386E) (Fig. 3C). Figure 3D demonstrates that ^mCherry^DDX6(WT) rescued the S387 phosphorylation-dependent binding of *Limk1* mRNA to Ago2, in contrast to ^mCherry^DDX6(R386E), which blocked this effect. These results indicate that DDX6 binding to RISC is required to mediate the S387 phosphorylation-dependent increase in *Limk1* mRNA binding to Ago2.

**Figure 4:**
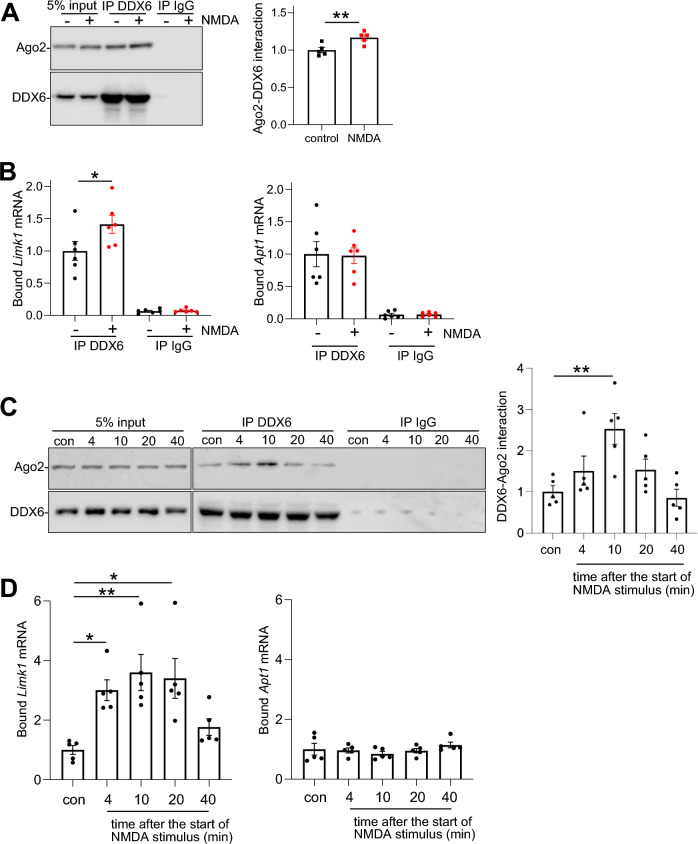
Rapid NMDAR-dependent increase in interaction of endogenous DDX6 with Ago2 protein and *Limk1* mRNA, but not *Apt1* mRNA. (**A**) NMDAR-dependent interaction of DDX6 with Ago2. Neurons were stimulated with NMDA or vehicle for 3 min, followed by 10 min incubation after NMDA washout. Cells were lysed and subjected to immunoprecipitation with anti-DDX6 or with control IgG. RNA and proteins were isolated by trizol extraction, bound mRNAs were quantified by qPCR and bound proteins were detected by Western blotting with anti-DDX6 or anti-Ago2. Left panel shows representative blots, graph shows quantification of Ago2-DDX6 interaction. n = 5 independent experiments, *p < 0.05, t-test. Data are mean ± S.E.M. (**B**) Quantification of *Limk1* and *Apt1* mRNA bound to DDX6 or to IgG control from IPs shown in A. Values are IP/input ratios, normalised to vehicle control. *p < 0.05, n = 6 independent experiments, two-way ANOVA followed by Tukey’s multiple comparison test. Data are mean ± S.E.M. (**C**) Ago2-DDX6 interaction increases rapidly after NMDAR stimulation. Neurons were stimulated with NMDA or vehicle for 3 min, followed by washout and a range of incubation times. The time points stated in the figure are the time after the start of NMDA application. Cells were lysed and treated as in A. Left panel shows representative Western blots, graph shows quantification of Ago2-DDX6 interaction over time. n = 5 independent experiments, *p < 0.05, **p < 0.01, one-way ANOVA followed by Tukey’s multiple comparison test. Data are mean ± S.E.M. (**D**) *Limk1*, but not *Apt1* mRNA shows rapid and transient increase in binding to DDX6 in response to NMDAR stimulation. Graphs show quantification of *Limk1* and *Apt1* mRNA bound to DDX6 at each time point after NMDAR stimulation. Values are IP/input ratios, normalised to vehicle control. n = 5 independent experiments, *p < 0.05, one-way ANOVA followed by Tukey’s multiple comparison test. Data are mean ± S.E.M.

To investigate the role of DDX6 further, we carried out DDX6 RIPs using DDX6-specific antibodies to analyse mRNAs associated with complexes containing endogenous DDX6 in response to NMDAR stimulation. Western blot analysis demonstrated efficient IP of DDX6, and NMDAR-dependent increase in association with Ago2 (Fig. 4A). qPCR analysis of RNA isolated by IP demonstrated that *Limk1*, but not *Apt1* mRNA binding to DDX6 increased 10 min after NMDAR stimulation (Fig. 4B). We extended this experiment over a 40 min time course, which demonstrated that a significant NMDAR-dependent increase in Ago2-DDX6 interaction occurs within 4 min after the start of NMDA stimulation and reaches a peak at around 10 min (Fig. 4C). Furthermore, *Limk1* mRNA binding to DDX6 followed a similar rapid and transient time course; a significant increase was observed at 4 min after the start of stimulation, maximal binding at 10 min, and a return to baseline was seen at 40 min (Fig. 4D). NMDAR stimulation had no significant effect on *Apt1* mRNA binding to DDX6 at any time point (Fig. 4D).

### NMDAR-dependent increase in binding of *Limk1* mRNA to Ago2 requires RISC-associated DDX6

**Figure 5:**
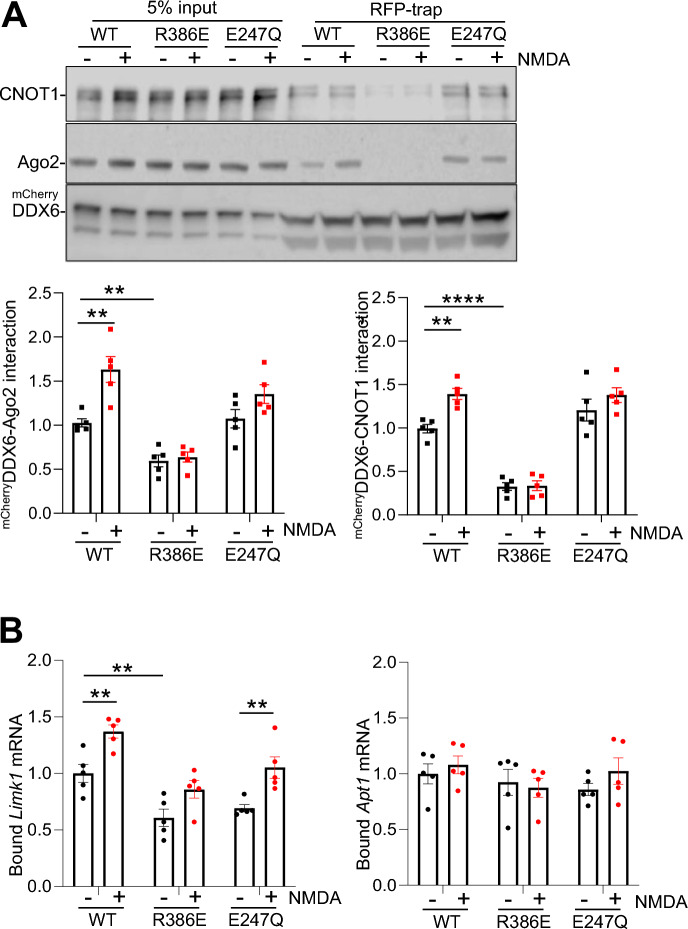
NMDAR-dependent increase in *Limk1* mRNA binding to DDX6 requires association with RISC. Neurons transduced with lentiviral vectors expressing mCherry, DDX6 shRNA and/or ^mCherry^DDX6(WT, R386E or E247Q) were stimulated with NMDA or vehicle for 3 min, followed by 10 min incubation after NMDA washout. Cells were lysed and incubated with RFP-trap beads. RNA and proteins were isolated by trizol extraction, bound mRNAs were quantified by qPCR and proteins were detected by Western blotting using anti-mCherry, anti-CNOT1 or anti-Ago2 antibodies. (**A**) R386E mutation blocks basal and NMDAR-dependent DDX6 interaction with CNOT1 and Ago2. Top panel shows representative blots, graphs show quantification of ^mCherry^DDX6-Ago2 and ^mCherry^DDX6-CNOT1 interactions. n = 5 independent experiments, *p < 0.05, two-way ANOVA followed by Tukey’s multiple comparison test. Data are mean ± S.E.M. (**B**) R386E mutation blocks basal and NMDAR-dependent *Limk1*, but not *Apt1* mRNA binding to DDX6. Graphs show quantification of *Limk1* and *Apt1* mRNA bound to ^Cherry^DDX6. Values are IP/input ratios, normalised to WT vehicle control. n = 6 independent experiments, *p < 0.05, two -way ANOVA followed by Tukey’s multiple comparison test. Data are mean ± S.E.M.

To investigate whether the NMDAR-dependent increase in *Limk1* binding to DDX6 requires its association with RISC, we analysed mRNA binding to ^mCherry^DDX6(R386E) compared to ^mCherry^DDX6(WT). Western blotting confirmed that the R386E mutation disrupts the ^mCherry^DDX6-CNOT1 interaction and hence ^mCherry^DDX6 association with Ago2 (Fig. 5A). Figure 5B shows that *Limk1* mRNA binding to DDX6 was markedly attenuated by the R386E mutation under basal conditions, whereas binding of *Apt1* mRNA to DDX6 was unaffected. Furthermore, disrupting DDX6 association with RISC by the R386E mutation blocked the NMDAR-dependent increases in *Limk1* mRNA binding to DDX6 and RISC protein–protein interactions (Fig. 5A and B).

In addition, since DDX6 is an ATP-dependent helicase, we investigated whether helicase activity is involved in the NMDAR-dependent protein–protein interactions or NMDAR-dependent binding of *Limk1* mRNA to RISC, by expressing DDX6 containing a well characterized mutation (E247Q) that blocks enzymatic activity^30,34^. The E247Q mutation had no significant effect on ^mCherry^DDX6- CNOT1 interactions under basal or stimulated conditions, but attenuated the NMDAR-dependent increase in ^mCherry^DDX6 association with Ago2 (Fig. 5A). The NMDAR-dependent increase in *Limk1* mRNA binding to DDX6 was not significantly affected by the E247Q mutation (Fig. 5B), suggesting that DDX6 helicase activity is not required for the association of *Limk1* mRNA with DDX6. Importantly, binding of *Apt1* mRNA to DDX6 was unaffected by NMDAR stimulation, E247Q mutation or R386E mutation (Fig. 5B), suggesting that silencing of *Apt1* occurs via a distinct mechanism.

Taken together, these data indicate that NMDAR-dependent Ago2 phosphorylation recruits DDX6 to RISC via CNOT1, which increases the binding of *Limk1*, but not *Apt1* mRNA to Ago2.

### DDX6 is required for NMDAR-dependent silencing of *Limk1*

**Figure 6:**
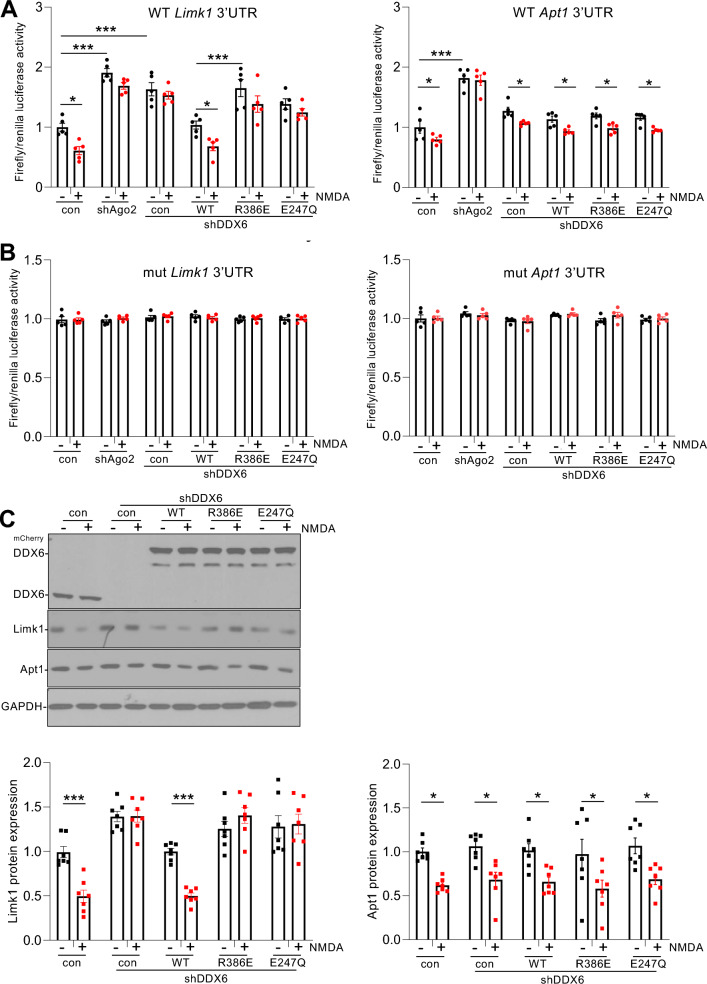
MiR-134-mediated NMDAR-dependent *Limk1* silencing requires DDX6. (**A**) NMDAR-dependent silencing via miR-134 and *Limk1* 3’UTR requires DDX6. Cultured neurons transfected with plasmids expressing mCherry, Ago2 shRNA, DDX6 shRNA, ^mCherry^DDX6(WT, R386E or E247Q) as shown, as well as *Firefly* luciferase reporters containing *Limk1* or *Apt1* WT 3’UTRs and *Renilla* luciferase, were treated with NMDA or vehicle for 3 min. 10 min after NMDA washout, cells were lysed and prepared for dual-luciferase assays. Graphs show *Firefly*/*Renilla* ratios, normalized to vehicle control; n = 5 independent experiments, *p < 0.05; **p < 0.01; ***p < 0.001; two-way ANOVA, followed by Tukey’s multiple comparison test. Data are mean ± S.E.M. (**B**) As for A, except luciferase reporters contained *Limk1* or *Apt1* 3’UTRs carrying mutations in the miR-134 or miR-138 seed sequences, respectively. (**C**) NMDAR-dependent down-regulation of Limk1 protein requires DDX6. Cultured neurons transduced with lentiviral vectors expressing mCherry, Ago2 shRNA, DDX6 shRNA, ^mCherry^DDX6(WT, R386E or E247Q) as shown were treated with NMDA or vehicle for 3 min. 10 min after NMDA washout, cells were lysed and prepared for Western blotting with anti-DDX6, anti-Limk1, anti-Apt1 or anti-GAPDH. Left panel shows representative blots, graphs show quantification of Limk1 and Apt1 expression, normalised to GAPDH. n = 7 independent experiments, *p < 0.05, two-way ANOVA followed by Tukey’s multiple comparison test. Data are mean ± S.E.M.

Our data presented so far suggest that DDX6 association with RISC is involved in the specific silencing of *Limk1* in response to NMDAR stimulation. While DDX6 has been shown to be required for miRNA-dependent silencing of reporters with artificial 3’UTRs harbouring multiple miRNA recognition elements (MREs) for the miRNA let-7^27,29,30^, it is unknown whether it has similar activity in all miRNA-dependent silencing or a greater effect on specific genes compared to others. To test the hypothesis that DDX6 association with RISC is required for NMDAR-dependent silencing of *Limk1*, we used dual-luciferase assays, with *Firefly* luciferase reporter constructs incorporating 3’UTRs of *Limk1* or *Apt1* and *Renilla* luciferase as control. In these assays, a decrease in *Firefly*/*Renilla* luciferase activity ratio represents an increase in miRNA-mediated gene silencing (and vice versa). We previously demonstrated NMDAR-dependent silencing of the *Limk1* reporter via miR-134 and of the *Apt1* reporter via miR-138 within 10 min after stimulation^16,35,36^, which is confirmed here (Fig. 6A). Knockdown of Ago2 by shRNA caused a significant increase in basal expression of both *Limk1* and *Apt1* reporters, confirming regulation by miRNA activity, and NMDAR stimulation had no effect in the presence of Ago2 shRNA, confirming that the effect of NMDA is miRNA-dependent. Interestingly, DDX6 knockdown had differential effects on *Limk1* and *Apt1* reporters under basal and NMDA-stimulated conditions, with *Limk1* showing a significant increase in basal expression and a block of NMDAR-dependent silencing, whereas *Apt1* was unaffected by DDX6 knockdown. ^mCherry^DDX6(WT) rescued *Limk1* reporter expression back to control levels, and restored NMDAR-dependent silencing. In contrast, replacing endogenous DDX6 with ^mCherry^DDX6(R386E) or ^mCherry^DDX6(E247Q) blocked NMDAR-dependent *Limk1* silencing to a similar extent as DDX6 shRNA alone (Fig. 6A). To confirm that the observed changes in reporter expression were miRNA-dependent, we performed parallel experiments with reporter constructs carrying mutations in the miR-134 or miR-138 seed regions of *Limk1* or *Apt1* 3’UTRs respectively, to prevent miRNA binding^16,37^. The seed region mutations abolished translational control by NMDAR stimulation and by DDX6 manipulations (Fig. 6B), supporting our conclusions that the observed changes are mediated by altered miR-134, and miR-138 activity, respectively.

To investigate the control of endogenous protein expression, we knocked down endogenous DDX6 and expressed shRNA-resistant ^mCherry^DDX6(WT, R386E or E247Q), and analysed NMDAR-dependent changes in Limk1 and Apt1 protein levels by Western blotting. We previously demonstrated that NMDAR stimulation causes a decrease in both Limk1 and Apt1 protein expression 30 min after stimulation, which is confirmed here (Fig. 6C;^16^). Consistent with our luciferase assay results, DDX6 knockdown blocked the NMDAR-dependent reduction in Limk1 protein, which was rescued by co-expression of ^mCherry^DDX6(WT) but not by ^mCherry^DDX6(R386E) or ^mCherry^DDX6(E247Q). In contrast, neither basal nor NMDAR-dependent Apt1 protein expression was affected by knockdown or mutation of DDX6 (Fig. 6C).

Taken together, these experiments demonstrate that recruiting DDX6 to RISC and DDX6 helicase activity are required for the rapid NMDAR-dependent silencing of *Limk1*, but not *Apt1*.

### DDX6 is essential for NMDAR-dependent dendritic spine shrinkage

**Figure 7:**
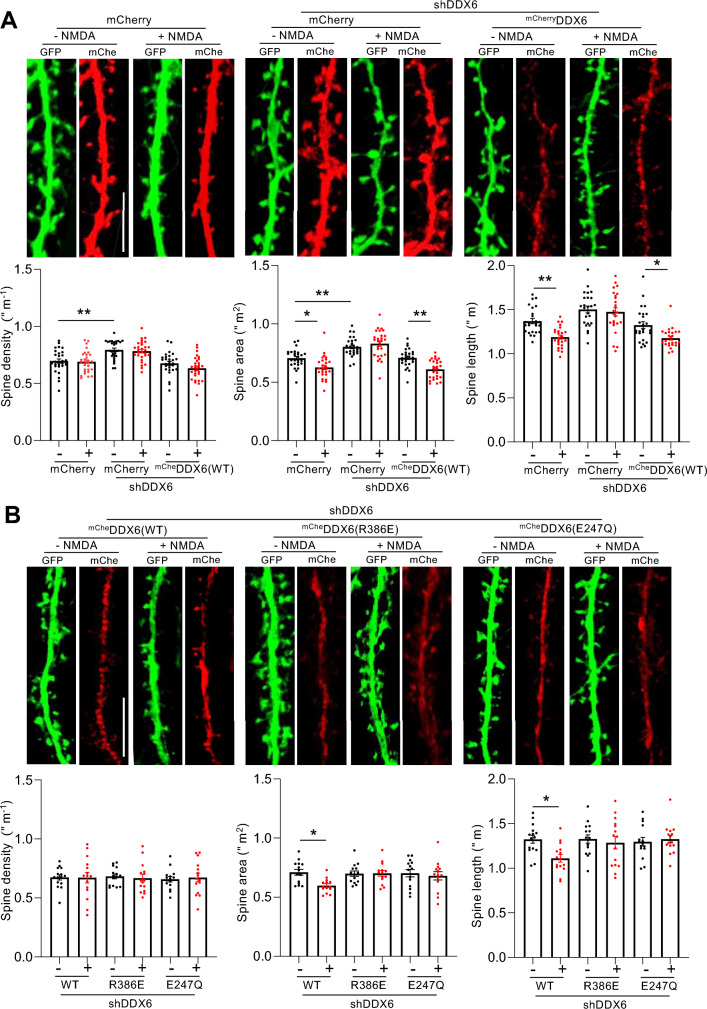
DDX6 is essential for NMDAR-dependent dendritic spine shrinkage. (**A**) DDX6 knockdown blocks NMDAR-dependent spine shrinkage. Neurons co-transfected with plasmids expressing GFP as a morphological marker and mCherry, DDX6 shRNA, ^mCherry^DDX6 as shown were treated with NMDA or vehicle for 3 min. 40 min after NMDA washout, cells were fixed and imaged by confocal microscopy, from which spine size and density were measured. Representative images of dendrites are shown. Graphs show quantification of spine size and spine density; n = 25–30 cells per condition (each data point corresponds to one cell) from 5 independent experiments, *p < 0.05; **p < 0.01; ***p < 0.001; one-way ANOVA followed by Tukey’s multiple comparison test. Scale bar = 10 μm. Data are mean ± S.E.M. (**B**) DDX6 helicase activity and association with RISC are required for NMDAR-dependent spine shrinkage. Neurons co-transfected with plasmids expressing GFP as a morphological marker and DDX6 shRNA plus ^mCherry^DDX6(WT, R386E or E247Q) were treated as in A. n = 14–16 cells per condition (each data point corresponds to one cell) from 4 independent experiments, *p < 0.05; **p < 0.01; ***p < 0.001; two-way ANOVA followed by Tukey’s multiple comparison test. Scale bar = 10 μm. Data are mean ± S.E.M.

We previously demonstrated that Ago2 phosphorylation at S387 is involved in regulating dendritic spine morphology via *Limk1* silencing^16^. Our results from the current study indicate that DDX6 is involved in NMDAR- and S387 phosphorylation-dependent silencing of *Limk1*, leading to the hypothesis that DDX6 plays a role in regulating spine morphology. To test this hypothesis, we analysed basal spine size and NMDA-stimulated dendritic spine shrinkage on neurons transfected with DDX6 shRNA with or without sh-resistant ^mCherry^DDX6, plus GFP as a morphological marker. Under basal conditions, DDX6 depletion by shRNA caused a small but significant increase in spine size, which was rescued by co-expression of sh-resistant ^mCherry^DDX6 (Fig. 7A). NMDAR stimulation caused significant spine shrinkage in control cells expressing GFP and mCherry at 30 min after stimulation. DDX6 depletion by shRNA completely blocked NMDAR-dependent spine shrinkage, which was rescued by co-expression of sh-resistant ^mCherry^DDX6(WT). We also analysed the number of spines per unit length of dendrite, and found that DDX6 knockdown caused a significant increase in basal spine density (Fig. 7A). Consistent with our previous work^16^, our NMDAR stimulation protocol did not cause a detectable change in spine density in any condition. We analysed spine morphology further, by classifying spines as mushroom, thin or stubby^38^. This analysis demonstrated that NMDAR stimulation caused a reduction in the percentage of mushroom spines and a corresponding increase in the percentage of stubby spines; these changes were blocked by DDX6 knockdown by shRNA. The percentage of thin spines was not significantly affected by NMDAR stimulation or DDX6 knockdown (Supplementary Fig. 2A).

In addition, we analysed the effect of co-expressing ^mCherry^DDX6(R386E or E247Q) with DDX6 shRNA. As shown in Fig. 7A, ^mCherry^DDX6(WT) rescued the effect of DDX6 knockdown, both on basal spine size and on NMDAR-dependent spine shrinkage (Fig. 7B). In contrast, neither ^mCherry^DDX6(R386E) nor ^mCherry^DDX6(E247Q) rescued the effect of DDX6 knockdown on spine size (Fig. 7B) or spine morphology (Supplementary Fig. 2B), indicating that DDX6 association with RISC, and DDX6 helicase activity are required for NMDA-induced spine shrinkage.

## Discussion

We have identified a novel mechanism for rapid and selective gene silencing in response to NMDAR stimulation. We previously demonstrated that NMDAR stimulation causes an increase in Ago2 phosphorylation at S387 to enhance silencing of *Limk1*^16^. Here, we elucidate the underlying mechanism by showing that NMDAR-dependent S387 phosphorylation causes an increase in binding of *Limk1*, but not *Apt1* mRNA with RISC within a few minutes of NMDAR stimulation. The phosphorylation-dependent binding of *Limk1* mRNA to Ago2 is abolished in the absence of RISC-associated DDX6, indicating that S387 phosphorylation does not affect the binding of mRNA to Ago2 directly, but instead requires DDX6. We propose a mechanism whereby the increased recruitment of DDX6 to RISC as a result of S387 phosphorylation generates a more favourable platform for binding specific mRNAs and hence for silencing. The previously-reported characteristic of DDX6 in binding secondary structures in mRNA 3’UTRs could play a role in this process^39–41^.

### A silencing mechanism for specific mRNAs

A key aspect of our findings is the specificity of the S387 phosphorylation/DDX6-dependent mechanism for *Limk1* in contrast to *Apt1*. We anticipate that *Limk1* is representative of a population of genes regulated via the same mechanism, and additional work will be needed to define this population via an unbiased screen. Our previous study demonstrated that *Firefly* luciferase reporters incorporating 3’UTRs of other miR-134 targets (*Pumilio2* and *Creb1*) are insensitive to regulation by S387 phosphorylation, indicating that the specificity for regulation lies in the 3’UTR of the target mRNA^16^. It has been demonstrated previously that DDX6 associates preferentially with specific mRNAs via stem loops or dumbbell structures in their 3’UTRs^39–41^; perhaps the existence of as yet unidentified secondary structures in the *Limk1* 3’UTR selects this mRNA for regulation by DDX6 following S387 phosphorylation of Ago2. Importantly, secondary structures in the 3’UTR can affect silencing by influencing miRNA accessibility, because miRNAs bind inefficiently to MREs located within double-stranded stem structures^42,43^. Hence, it is possible that the structural elements that promote association with RISC via DDX6 also limit miRNA activity until DDX6 binds and unmasks the MRE via its helicase activity. Further work will be needed to elucidate the precise role of DDX6 helicase activity in regulating mRNA 3’UTR structure and consequent miRNA activity.

We anticipate that the novel mechanism described here is part of a complex process for defining targets for miRNA-dependent silencing at synapses, with additional factors such as the spatial distribution of relevant mRNAs and miRNAs playing an essential complementary role. In neurons, specific mRNAs and miRNAs (including *Apt1* mRNA, miR-138, *Limk1* mRNA and miR-134) are targeted to dendrites to maintain a local pool for translational control of synaptic protein synthesis^22,24^. The increased availability of these spatially targeted mRNAs/miRNAs will strongly influence the pool of gene silencing events available for rapid regulation by NMDAR stimulation. We propose that the miRNA-dependent silencing of this pool is further refined (to include *Limk1*, but exclude *Apt1*) in response to NMDAR stimulation by the pS387/DDX6-dependent mechanism described here.

It has been shown previously that Ago2 phosphorylation at S387 also recruits DDX6 to RISC in non-neuronal cells that would require different patterns of gene silencing^32^. In the absence of the extended arborised morphology of neurons, different local targeting and hence spatial distributions of mRNAs/miRNAs, as well as different upstream signalling to regulate S387 phosphorylation would be involved. Hence, we anticipate that the S387 phosphorylation/ DDX6-dependent mechanism described here would mediate distinct programmes of gene silencing in different cell types.

We anticipate that *Apt1* is representative of a pool of genes whose silencing is modulated by NMDAR stimulation, but via a distinct mechanism that requires Ago2, but does not involve S387 phosphorylation or DDX6. Our results suggest an additional mode of NMDAR-dependent silencing for *Apt1*, perhaps whereby NMDAR stimulation promotes the association of additional regulatory proteins with the RISC to increase silencing efficiency, while leaving Ago2 binding to *Apt1* mRNA unaffected.

Decapping proteins such as DCP1/2 and deadenylation proteins including PAN2-PAN3 and CCR4-NOT that regulate translational repression, associate with Ago2 in the RISC via scaffold proteins GW182 and CNOT1^44^. While the regulation of these protein–protein interactions by NMDAR stimulation has not, to our knowledge, been studied, it is possible that they could be increased following stimulation to promote silencing independently of S387 phosphorylation and DDX6.

The existence of different NMDAR-dependent mechanisms modulating distinct programmes of gene silencing events suggests additional levels of upstream signaling are involved to define which mechanism is activated. For example, the magnitude or kinetics of the NMDAR stimulus, or the NMDAR subunit composition involved might determine which pathway is recruited. In addition, various neuromodulators are involved in memory processes; for example, cholinergic activation of muscarinic receptors modulates plasticity at excitatory synapses and is essential for specific forms of memory^45^. Interestingly, cholinergic stimulation can regulate the activity of p38 MAP kinase and of Akt^46,47^, both of which mediate Ago2 phosphorylation at S387^48,49^.

### Multiple potential mechanisms for regulating miRNA-dependent gene silencing

Numerous mechanisms are potentially available for regulating miRNA-dependent gene silencing, including transcriptional control of miRNA gene expression to regulate the availability of specific miRNAs; spatial targeting of RNAs to dendrites; post-translational modifications of Ago2 and other components of the RISC or RISC loading complex (RLC) to modulate protein-RNA interactions directly; and post-translational modifications to regulate RISC or RLC protein–protein interactions.

Considering each of these in turn, while it has been shown that miRNA gene transcription is regulated by NMDAR stimulation^14,15^, for processes where miRNA-dependent silencing is required within a few minutes of the stimulus, transcriptional control is not fast enough. This is especially the case in neurons where the location of the stimulus, and of the consequent silencing, may be far from the cell nucleus and would rely on long-distance RNA transport along dendrites. While it has been shown that specific miRNAs essential for NMDAR-dependent plasticity are transcriptionally regulated in response to stimulation^14,50,51^, perhaps the role of increased transcription is to replenish the pool of miRNAs used locally at dendritic spines in rapid response to stimulation. It has been shown that a cluster of phosphorylation sites at the C-terminus of Ago2 can modulate Ago2-mRNA interactions^52^, and the same cluster has recently been shown to regulate Ago2 release from mRNA^53^. While phosphorylation of these sites holds potential as a mechanism for rapid regulation of silencing in neurons, to our knowledge, there are no reports of such phosphorylation in response to neuronal stimulation.

In addition, our data suggest additional levels of RISC regulation by NMDAR stimulation, suggesting that as yet unidentified mechanisms may also be involved. For example, the DDX6 helicase mutant (E247Q) blocked the NMDAR-dependent increase in Ago2-DDX6 interaction, but the same mutation did not attenuate the NMDAR-dependent increase in *Limk1* mRNA binding to DDX6. This suggests that the NMDAR-dependent recruitment of DDX6 to RISC requires DDX6 helicase activity, as well as phosphorylation of Ago2 at S387.

The mechanism we describe here, whereby phosphorylation of Ago2 and consequent increase in DDX6 interaction with RISC promotes binding to specific mRNAs, represents an efficient mechanism for modulating miRNA-dependent silencing within a few minutes of NMDAR stimulation. We anticipate that additional protein–protein interactions in RISC might also be regulated by NMDAR stimulation to facilitate this process.

### DDX6 as a critical player in dendritic spine plasticity

Our data demonstrate a critical role for DDX6 in mediating NMDAR-dependent spine shrinkage; both DDX6 helicase activity and its association with RISC are necessary for spine shrinkage, which is consistent with our biochemical data indicating that these properties of DDX6 are necessary for NMDAR-dependent silencing of *Limk1*. Limk1 protein promotes actin polymerisation and actin filament stability, and it has been demonstrated previously that loss of Limk1 activity results in actin depolymerisation in dendritic spines and consequent spine shrinkage^54,55^.

We detected an increase in colocalisation of DDX6 with Ago2 in neuronal dendrites in response to NMDAR stimulation, but not in cell bodies, supporting an important functional role for DDX6 in plasticity. Furthermore, dendritic DDX6 increased in response to NMDAR stimulation, with a concomitant decrease in cell body levels, consistent with a translocation of DDX6 protein from cell bodies towards dendrites. A recent report described changes in DDX6 localisation in neuronal cell bodies in response to NMDAR stimulation^56^.

The study focussed on RNA granules, which are membraneless condensates of RNAs and proteins (including DDX6 and other components of the miRNA machinery) involved in regulating mRNA fate, and consequently modulate various aspects of protein expression. Dendrites were not analysed in Bauer et al., whose analysis appeared to focus on cell bodies, where DDX6-containing granules were found to be more prevalent in immature neurons and to disassemble during neuronal maturation. Furthermore, NMDAR stimulation decreased DDX6 clustering in cell body granules^56^, which is consistent with the translocation we observe here. Perhaps the NMDAR-dependent increase in Ago2-DDX6 colocalisation we observe in dendrites is caused by both translocation of DDX6 from cell body to dendrite and enhanced Ago2 S387 phosphorylation to promote association of DDX6 with RISC. Further work is needed to elucidate the mechanistic details that underpin this process.

In conclusion, this work has defined a mechanism for the selective miRNA-dependent silencing of *Limk1* in response to NMDAR stimulation that is essential for dendritic spine plasticity. It will be intriguing to establish whether this DDX6-dependent mechanism functions to orchestrate a programme of gene silencing events essential for NMDAR-dependent plasticity in neurons. Furthermore, DDX6 mutations have recently been shown to cause intellectual disability, demonstrating a critical role for DDX6 in human brain function. We anticipate that regulation of RISC protein–protein interactions represent potential for therapeutic intervention in neurological disorders.

## Materials and methods

### Plasmid constructs

DDX6 knockdown and molecular replacement constructs were created by ligating sequences corresponding to DDX6 shRNA and sh-resistant DDX6 cDNA into pXLG3-PX-mCherry-WPRE. Oligos for shDDX6, Fwd: GATCCCCGATGATCGCTTCAACCTGATTCAAGAGATCAGGTTGAAGCGATCATCTTTTTC; Rev: TCGAGAAAAAGATGATCGCTTCAACCTGACTCTTGAATCAGGTTGAAGCGATCATCGGG were annealed in a thermocycler and ligated into pSUPER-neo-GFP via BglII and XhoI sites. The H1-shRNA cassette was then amplified by PCR using the generic primers H1_Pac1_F (CACTTAATT AACGAACGCTGACGTCATCAACC) and m13_30nt_R (AGCGGATAACAATTTCACACAGGA) and cloned into the PacI and XhoI sites of pXLG3-PX-mCherry-WPRE. ShRNA-resistant DDX6 was generated by introducing silent mutations by site-directed mutagenesis; Fwd primer: ATCAACTTGATCACATATGATGACCGGTTCAATCTGAAAAGTATTGAGGAGC; Rev primer: GCTCCTCAATACTTTTCAGATTGAACCGGTCATCATATGTGATCAAGTTGAT). DDX6 mutations were introduced by site-directed mutagenesis; E247Q Fwd: CAGATGATAGTATTGGATCAGGCAGATAAGTTGTTGTCA; Rev: TGACAACAACTTATCTGCCTGATCCAATACTATCATCTG). R386E Fwd: TTCCGAAATGGCTTATGCGAGAATCTTGTTTGCACTGAT; Rev: ATCAGTGCAAACAAGATTCTCGCATAAGCCATTTCGGAA). Ago2 constructs and Firefly Luciferase constructs carrying *Limk1* or *Apt1* 3′UTRs were described previously^16^. Plasmids are available on request to the corresponding author.

### Cortical neuronal cultures

Rat embryonic cortical neuronal cultures were prepared from E18 Wistar rat embryos of either sex (Charles River), as described previously^16^. All animal procedures relating to this study were approved by the Animal Welfare and Ethics Review Board at the University of Bristol. The study was conducted in accordance with the local legislation and institutional requirements and animals were sacrificed according to Home Office Schedule 1 regulations. All methods are reported in accordance with ARRIVE guidelines (https://arriveguidelines.org). Neurons were initially plated in plating medium (Neurobasal (Gibco) supplemented with B27 (Gibco), 5% horse serum and 5 mM Glutamax), which was changed to feeding medium (Neurobasal supplemented with B27 and 2 mM Glutamax) two hours after plating. One-third of the culture medium volume was removed and replaced with fresh medium at 6 and 12 days in vitro (DIV). Neurons were treated with Fluorodeoxyuridine to inhibit glial cell proliferation at DIV 6. Neurons were plated at densities of 100,000 cells per well of a 24-well plate for luciferase assays; 400,000 cells per well of a 6-well plate or 3,000,000 cells per 10 cm dish for biochemistry experiments; 120,000 cells per 22 mm coverslip coated in Poly-D-lysine in 6-well plates for imaging experiments. Neurons in 6 well/24 well plates were transfected with a total of 2 μg/1 μg plasmid DNA at 10–12 DIV using 3 μl/1.5 μl Lipofectamine 2000 (Invitrogen) per well and used for experiments at 17–21 DIV. Lentiviral transductions were carried out at DIV 10–12, and cultures used for experiments at 17–21 DIV. NMDAR stimulation was by bath application of 50 μM NMDA (Tocris) plus 20 μM glycine in HBS (140 mM NaCl, 5 mM KCl, 25 mM HEPES pH 7.4, 1.8 mM CaCl_2_, 0.8 mM MgCl_2_ 10 mM glucose) for 3 min at 37 °C, followed by two washes in HBS lacking NMDA and subsequent incubation in HBS for 10 min (unless otherwise stated). Neurons were then harvested or fixed at specific time points as stated for biochemistry, luciferase assays or imaging.

### Confocal microscopy and image analysis

Neurons grown on coverslips were fixed in 4% paraformaldehyde (ThermoFisher) in PBS supplemented with 4% sucrose at RT for 15 min. After washing with PBS, unreacted PFA was quenched with 100 mM glycine for 5 min. Cells were then permeabilised for 4 min in 0.1% Triton X-100 and blocked in 3% BSA for 20 min. Coverslips were incubated with anti-DDX6 (Sigma, 1:300 dilution ) and Anti-Ago2 (Sigma,1:400 dilution) in 5% BSA for 1 h, washed in PBS and incubated with appropriate secondary antibodies (AlexaFluor 488, 568, 647; Life technologies or Millipore) for 1 h, and mounted on slides in Fluoromount. Coverslips were imaged on a Leica DMI 6000 SP5-II confocal system under a 63 × /1.4 NA oil-immersion lens at a resolution of 2024 × 2024 pixels, pixel size 60 nm. The experimenter was blinded to the experimental condition. For spines analysis, three secondary dendrites with lengths greater than 50 μm were selected per neuron using GFP signal as the morphological marker. 100–150 spines were analysed per neuron; 549–638 spines were analysed per condition. We acquired stacks of 4–5 z-planes (1.2 μm thick) and generated a maximum intensity projection in ImageJ. The spine measurements were performed in ImageJ by converting the GFP signal to a binary mask. The “Polygon selection” tool was used to define a region of interest (ROI) containing and isolating all the spines along one side of the dendrite in the 2D image. The “Analyse particles” tool was then used to calculate the area of each spine within the ROI as well as the number of spines. We set a size inclusion range of 0.1 – 4 μm^2^. This was repeated for the other side of the dendrite and the data pooled for the whole dendrite. Spine lengths were measured using the line tool. For colocalization analysis, single planes were acquired and three secondary dendrites were analysed per cell. The “Coloc2” plugin in ImageJ was used to quantify Pearson’s colocalization coefficient (PCC). To control for random colocalization, we performed 100 Costes randomisations^57^ in the Coloc2 plugin, which demonstrated that no randomised images produced PCC values greater than the actual PCC value. Levels of DDX6 in dendrites were quantified by measuring integrated densities of fluorescence signals in three dendrites per neuron.

For analysis of spine morphology, 4–6 neurons were analysed from each of 4–5 independent experiments to generate a sample of n = 16–30. For immunocytochemistry experiments, 4–6 neurons were analysed from each of 4–5 independent experiments, but n = 4–5, i.e. each data point represents one coverslip from one dissection, to account for batch effects between the different neuron preparations. Power calculations, based on previous similar experiments^16^, were used to define appropriate sample sizes. Statistical significance was determined using t-test for two-condition (control vs NMDA) experiments or one-way ANOVA followed by Tukey’s post-hoc test for experiments with multiple conditions (control vs NMDA combined with three different transfection conditions).

### Luciferase assays

DIV12 cortical cultures were co-transfected with *Firefly* luciferase reporter constructs incorporating *Limk1* or *Apt1* 3’UTRs, *Renilla* luciferase control and pXLG vectors for manipulation of Ago2 or DDX6 expression. Dual-luciferase reporter assay system (Promega) was used to perform the assays according to the manufacturer's instructions. Readings were taken in duplicate from which the mean was calculated to give the value for each independent experiment. *Firefly/Renilla* ratios were calculated and normalised to control conditions in each of at least five independent experiments, and statistical significance was determined using two-way ANOVA followed by Tukey’s post-hoc test. Power calculations, based on previous similar experiments^16^, were used to define appropriate sample sizes.

### RNA-immunoprecipitations (RNA-IPs) using purified antibodies

All RNA-IP reagents were prepared in DEPC treated water and experiments were performed in a designated clean environment treated with RNaseZap (Thermo Fisher). All pipette tips and microfuge tubes were autoclaved before use.

Cells were lysed in IP buffer (0.5% Triton X-100, 150 mM NaCl, 20 mM HEPES, pH 7.4, RNase inhibitor (Thermo Fisher), Protease inhibitor (Roche), phosphatase inhibitor cocktail 1 and 2 (Thermo Fisher) and 5% of lysate was removed for RNA and protein input. Lysates were incubated with 4 μg of anti-DDX6 (BioLegend, 674402), anti-Ago2 (FUJIFILM Wako Pure Chemical Corporation, 014-22023) or control IgG (Millipore 12-370) for 3 h at 4 °C followed by addition of protein G-sepharose beads (GE Healthcare) at 4 °C for 1 h. Beads were washed four times (1 min each with slow rotation) in 1 ml IP buffer at 4 °C. Bound RNA was analysed by qPCR and proteins were detected by Western blotting.

### RNA-IP using GFP/RFP-trap

GFP/RFP-trap (Chromotek) pull-downs were performed after lentiviral transduction of DIV10 cortical neurons with pXLG-^GFP^Ago2 or pXLG-^mCherry^DDX6 constructs. Cells were lysed 7 days later in 1 ml lysis buffer (0.5% Triton X-100, 150 mM NaCl, 10 mM Tris pH 7.5, 0.5 mM EDTA, RNase inhibitor (ThermoFisher), Protease inhibitor (Roche) and phosphatase inhibitor cocktail 1 and 2 (Thermo Scientific) on ice. 5% was taken for RNA and protein input. Lysates were incubated with 20 μl GFP-trap beads for 1 h at 4 °C. The beads were washed with 500 μl of wash buffer (1xPBS prepared in DEPC treated water) four times (1 min each with slow rotation) at 4 °C. Bound RNA was analysed by qPCR and proteins were detected by Western blotting.

### Real-time qPCR analysis

Total RNA was extracted using TRIzol reagent (Ambion, ThermoFisher Scientific) and reverse transcription was performed using RevertAid First Strand cDNA Synthesis Kit (ThermoFisher Scientific) according to the manufacturer’s instructions. Luciferase mRNA (Promega) was added as a spike-in control during the RNA extraction. Real time PCR was conducted using the MxPro™-Mx3000P qPCR system (Stratagene) and PowerUp™ SYBR™ Green Master Mix (Applied Biosystems, Thermo Fisher Scientific). Each measurement was performed in triplicate, from which the mean was calculated to give the value for each independent experiment. For each qPCR, a dissociation curve analysis was conducted. The primers used are listed in the Table below. The relative expression was analysed by normalising to their respective luciferase gene Ct value and then subsequently normalising to the input Ct value. These values were finally normalised to the Western blot signal. Statistical significance was determined using t-test for two-condition (control vs NMDA) experiments, or two-way ANOVA followed by Tukey’s post-hoc test for experiments with multiple conditions (e.g. control vs NMDA or control vs DDX6 shRNA combined with three different transfection conditions). Primers used for qPCR were as follows:

Limk1_fwd1: GGTTGACGCTACTTTGTTGC; Limk1_Rev1: AGATACTCTGGCTACAGCTCG;

Limk1-fwd2: AGTGCATGAGGTTGACGCTA; Limk1-Rev2: AGTGCATGAGGTTGACGCTA;

Apt1_fwd1: CGCCGCGGTTATTTTCCTTC; Apt1_Rev1: GGCGCATGTGGACAGATGTA;

Apt1_fwd2: CCGCGGTTATTTTCCTTCACG; Apt1_Rev2: TAACAGGCGCATGTGGACAGA;

Luciferase_Fwd: AGAGATACGCCCTGGTTCCT; Luciferase_Rev: ATAAATAACGCGCCCAACAC.

### Western blotting

Proteins were separated by sodium dodecyl sulfate–polyacrylamide gel electrophoresis (SDS-PAGE; 8–12%) and transferred onto polyvinylidene fluoride membranes (Merck). The membranes were blocked in 5% milk solution or 5% BSA made up in PBS-Tween-20 (PBST) for 1 h at RT. Subsequently, the membranes were incubated in primary antibodies diluted in 3% milk or 3% BSA in PBST overnight at 4 °C. Secondary antibodies conjugated to HRP were from GE Healthcare and used at 1:10,000 for 1 h at RT. The bands were visualised using ECL Western blotting substrates (ThermoFisher Scientific or GE Healthcare) and the Odyssey Fc-detection system (LI-COR). Western blot band intensities were analysed in Image studio Lite followed by appropriate statistical analysis. For GFP-trap and co-IPs, bound proteins were normalised to their respective inputs. Power calculations, based on previous similar experiments^16^, were used to define appropriate sample sizes. Statistical significance was determined using t-test for two-condition (control vs NMDA) experiments, or two-way ANOVA followed by Tukey’s post-hoc test for experiments with multiple conditions (e.g. control vs NMDA or control vs DDX6 shRNA combined with three different transfection conditions). The antibodies used for Western blotting, including dilutions, were as follows: Ago2 (C34C6; Cell Signaling Technology; 1:500 and 67934; Proteintech; 1:1000); DDX6 (A300-461A; Bethyl Laboratories; 1:2000); CNOT1 (D5M1K; Cell Signaling Technology; 1:1000); Limk1 (A302-670A; Bethyl Laboratories; 1:1000); GFP (PABG1; ChromoTek; 1:1000); mCherry (5F8; ChromoTek; 1:2000); Apt1 (Bethyl Laboratories; 1:1000); GAPDH (Ab8245; Abcam; 1:10000). Uncropped blots are presented in Supplementary Fig. 3.

### Supplementary Information

Supplementary Figures.

## Data Availability

The datasets generated during and/or analysed during the current study are available from the corresponding author on reasonable request.
