# Supplementary material for: An essential role for the RNA helicase DDX6 in NMDA receptor-dependent gene silencing and dendritic spine shrinkage
Source: Sci Rep. 2024 Feb 6;14:3066. doi: 10.1038/s41598-024-53484-4 (PMC10847504; doi:10.1038/s41598-024-53484-4)

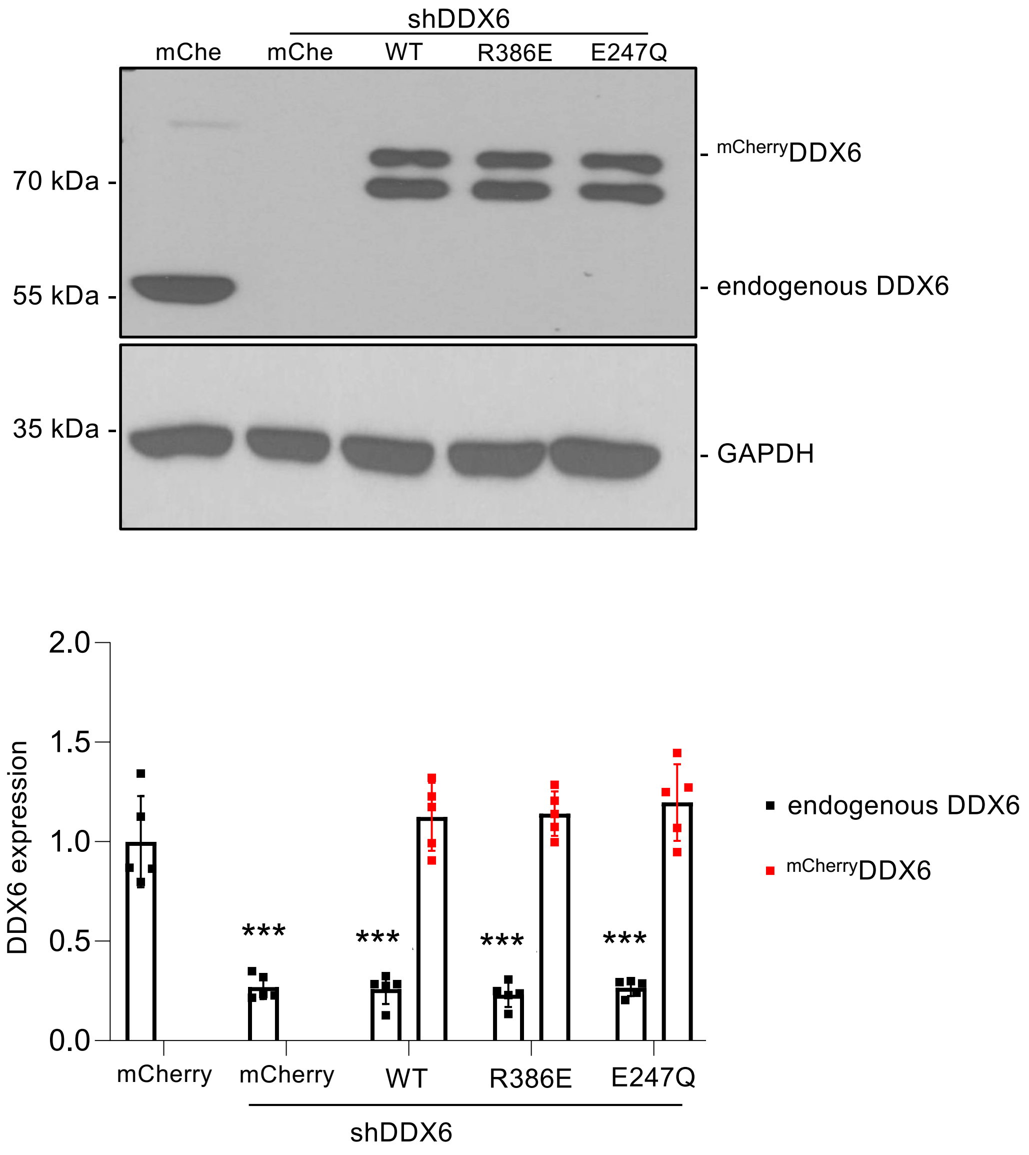

Supplementary figure 1

Characterisation of shRNA-mediated knockdown of DDX6 and replacement with recombinant ^{mCherry}DDX6.

Cortical neurons were transduced at DIV10 with lentiviruses expressing mCherry alone, mCherry plus DDX6 shRNA, DDX6 shRNA plus ^{mCherry}DDX6(WT, R386E or E247Q) as shown. 7 days later, cells were lysed and proteins analysed by Western blotting using anti-DDX6 or anti-GAPDH. Representative blots are shown.

^{mCherry}DDX6 runs as a doublet in anti-DDX6 blots, with the upper band corresponding to the predicted MW of the recombinant protein. In many cases, the upper band predominates (see Figs 5A and 6C), suggesting the lower band is a degradation product generated after cell lysis.

Graph shows DDX6 expression normalised to GAPDH. n=5, ***p<0.001, two-way ANOVA followed by Tukey's multiple comparison test.

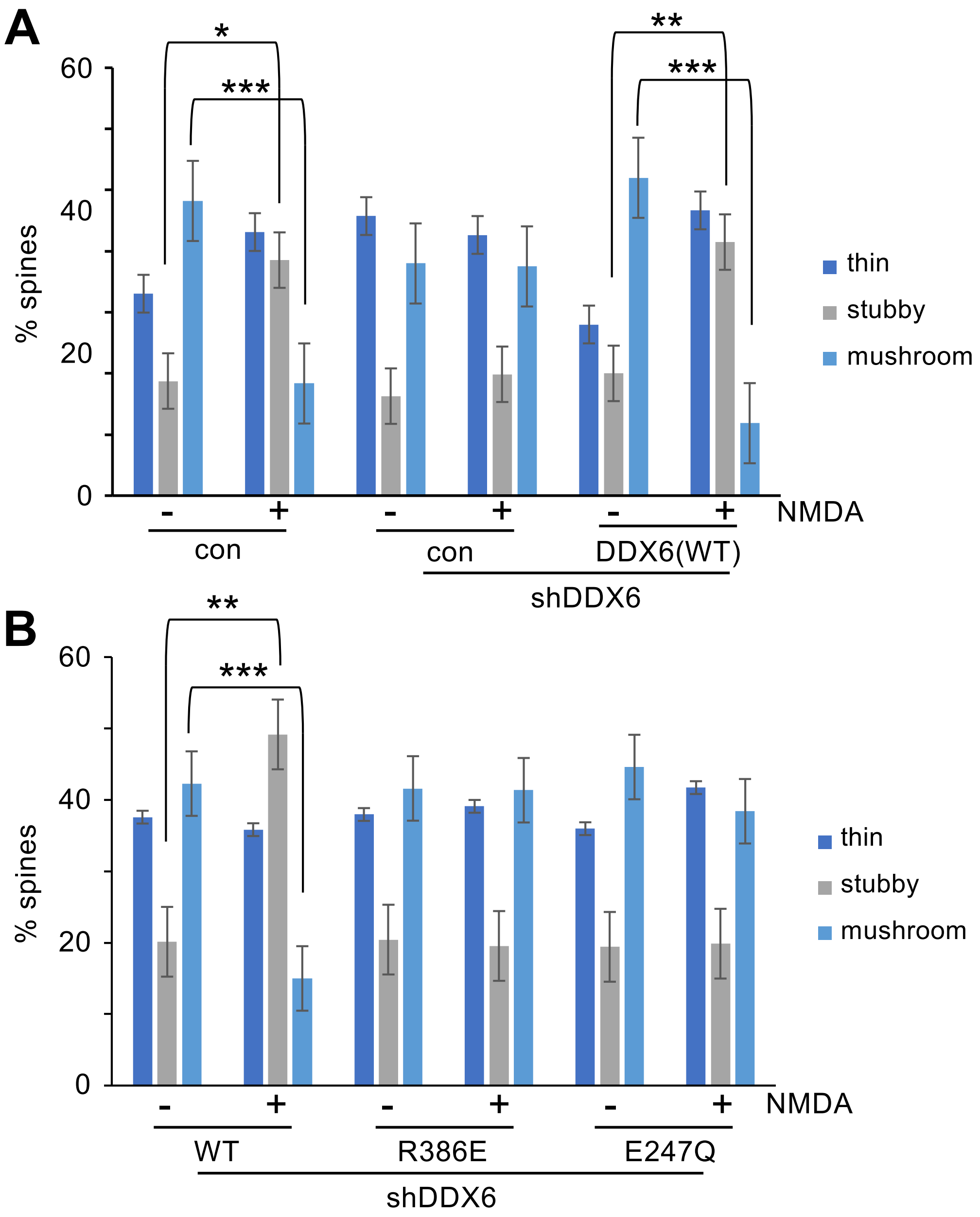

Supplementary figure 2

Classification of spine subtypes under conditions shown in Figure 7.

Spines were classified according to the following criteria:

Thin spines: head width > neck width, length > head width and length > 1 μm.

Mushroom spines: head width ≫ neck width and head width > 0.6 μm.

Stubby spines: head width ≅ neck width and length ≤ width.

Graphs show percentage of each spine type in the same dendritic segments analysed in Fig. 7.

A) Neurons co-transfected with plasmids expressing GFP as a morphological marker and mCherry, DDX6 shRNA, ^{mCherry}DDX6 as shown were treated with NMDA or vehicle for 3 min. 40 min after NMDA washout, cells were fixed and imaged by confocal microscopy, from which spines were analysed.

B) Neurons co-transfected with plasmids expressing GFP as a morphological marker and DDX6 shRNA plus ^{mCherry}DDX6(WT, R386E or E247Q) were treated as in A.

*p<0.05, **p<0.01, ***p<0.001; two-way ANOVA followed by Tukey's multiple comparison test. Data are mean ± SEM.

Supplementary Figure 3: Uncropped versions of blots presented in this study

Figure 1A

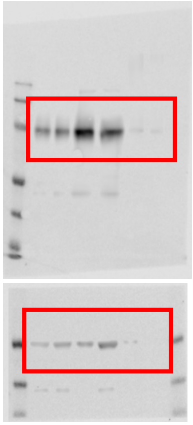

Figure 1C

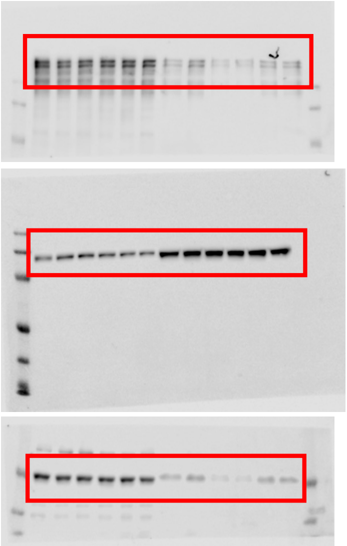

Figure 3A

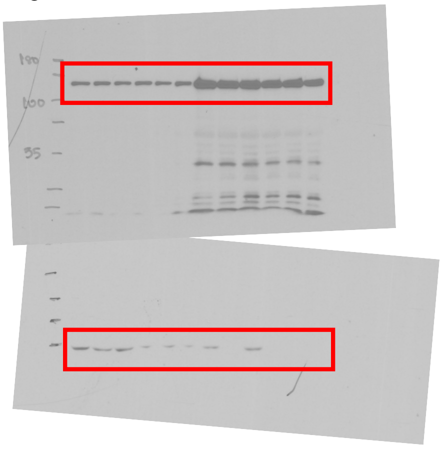

Figure 3C

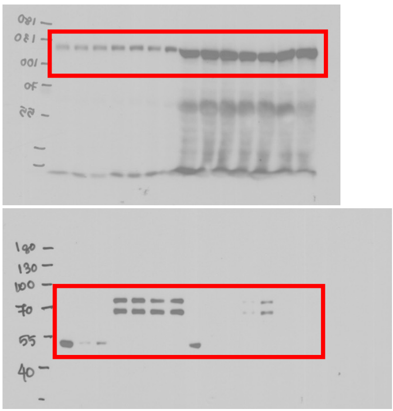

Figure 4A

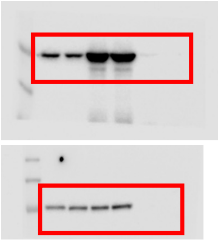

Figure 4C

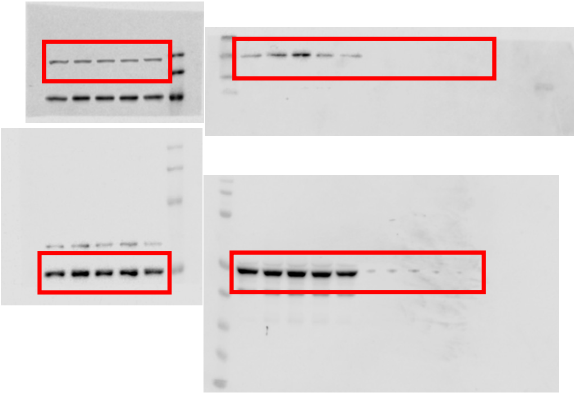

Figure 5A

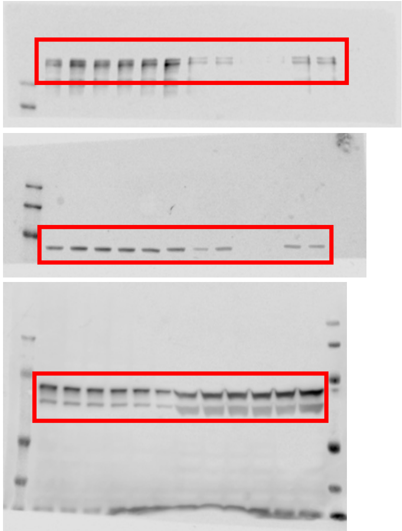

Figure 6C

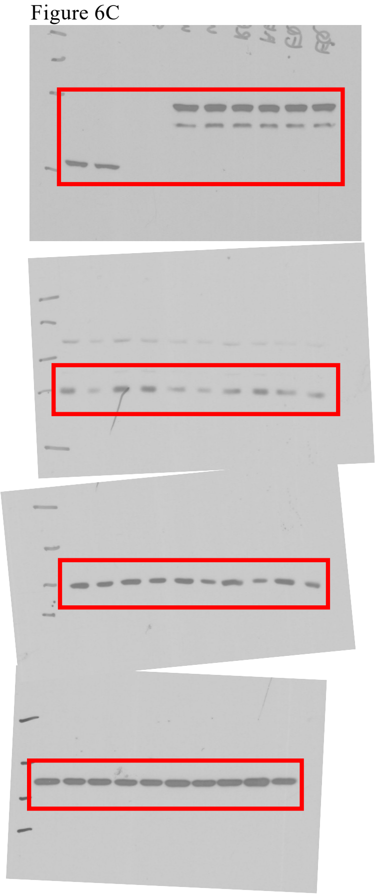

Supplementary Fig 1

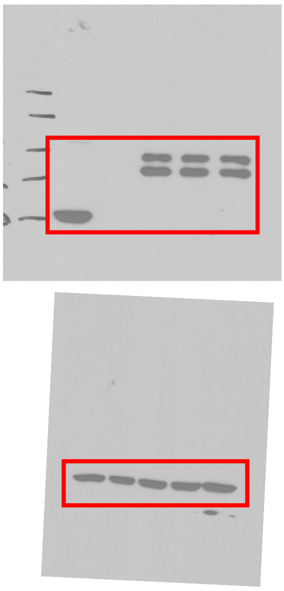

Supplement: Supplementary file 1 — Supplementary Figures. [file 41598_2024_53484_MOESM1_ESM.pdf]
